# Bilateral anterior congenital radial head dislocation in adults: a case report and literature review

**DOI:** 10.3389/fsurg.2023.1155461

**Published:** 2023-05-17

**Authors:** Jiahao Gao, Jinshuo Tang, Menglong Li, Haitao Li, Yachen Peng, Chenyu Wang, Tong Liu, Jianlin Zuo

**Affiliations:** Department of Orthopeadics, China-Japan Union Hospital, Jilin University, Changchun, China

**Keywords:** congenital radial head dislocation, ulnar osteotomy, open reduction, annular ligament reconstruction, case report

## Abstract

**Objective:**

Congenital dislocation of the radial head (CRHD) is a rare condition, with bilateral anterior cases being even less common worldwide. Only a few cases had residual pain after adulthood, even when left untreated. Herein, we describe an adult case of bilateral anterior CRHD with significant pain and snapping during motion. The aim of this study was to report the physical and radiological findings, treatment methods, and short-term outcomes of our case and to review adult CRHD cases in the literature.

**Patient:**

A 21-year-old male patient presented to our hospital with chief complaints of snapping and exacerbated pain during motion in his left elbow.

**Diagnoses and interventions:**

Detailed medical history and physical examination results were recorded. Radiographic examinations were performed on the bilateral elbow, and the diagnosis of bilateral anterior congenital radial head dislocation was confirmed. To relieve the pain and snapping in the left elbow, we performed open reduction and fixation of the radial head with annular ligament reconstruction and ulnar osteotomy. Postoperatively, the elbow rested at 90° flexion with a cast for 16 weeks, and the K-wire was removed on the 10th week; afterward, active functional exercises were performed.

**Outcomes:**

The patient was followed-up for 1 year. The pain in his left elbow was relieved with a reduction in the visual analog scale score from 7 to 3. The range of motion of the left elbow was changed from 0° to 135° (preoperative) to −5° to 120° (postoperative) (extension–flexion) without any snapping. However, restrictions in external rotation have not yet been fully resolved. Further physical rehabilitation is required.

**Conclusion:**

When managing patients with congenital radial head dislocation, the contralateral elbow should be evaluated to identify potential bilateral cases. Surgical options should be discussed with adult patients only for the strong need for functional improvement, although the outcomes may not be fully satisfactory.

## Introduction

Congenital dislocation of the radial head (CRHD) is a rare condition but is still the most common congenital abnormality of the elbow (1, 2). The estimated incidence of CRHD ranges from 0.06% to 0.16% (3–5). Unilateral or bilateral sides can be affected; sometimes, they are also associated with other genetic disorders (1, 6). One study suggested that CRHD is not commonly found at birth; rather, it is diagnosed in the patients’ childhood or adolescence when the limitation of range of motion (ROM) or pain in the elbow appears (7). Early diagnosis of this deformity is difficult because identification on plain radiographs in young children is hard. The epiphyseal centers of the radial head and capitellum of the humerus are immature, and the ossifications are not complete until 5 years of age (7). However, even if left untreated, adult CRHD cases with obvious symptoms are rare. Here, we describe an adult patient with progressive pain and snapping in his left elbow and discuss our treatment choices and the subsequent outcomes. Furthermore, we reviewed the literature regarding adult CRHD cases and summarized the progress in its etiology, diagnosis, and treatment.

## Case description

**Figure 1:**
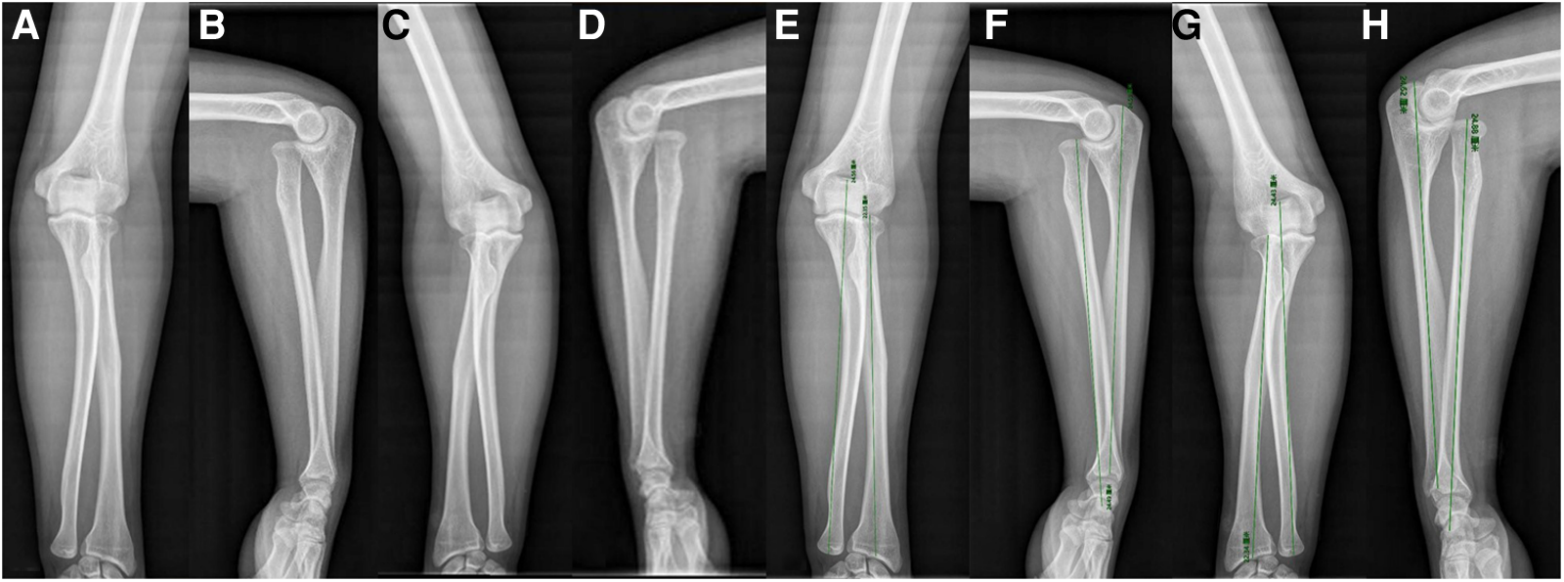
AP and LAT radiographs of bilateral anterior congenital radial head dislocation in a 21-year-old male. (**A**, **B**) AP and LAT of the left elbow. (**C**, **D**) AP and LAT of the right elbow. (**E**, **F**) AP and LAT showing the measurement of LRU of the left elbow; the RLRU are 0.9100 AP and 0.9984 LAT. (**G**, **H**) AP and LAT showing the measurement of LRU of the right elbow; the RLRU are 0.9144 AP and 1.0105 LAT. AP, anterior -posterior; LAT, lateral; LRU,the length of the radius and ulna; RLRU,the ratio of the length of radius to ulna.

This study was approved by the Ethics Committee of the China-Japan Union Hospital of Jilin University. Signed informed consent was obtained from the patient in accordance with the Declaration of Helsinki. A 21-year-old male patient presented to our hospital with the primary concern of increased pain and snapping during flexion when performing certain rotating actions, such as turning a key in a door or flexing his left elbow. He had no history of trauma, relevant family history, or other congenital abnormalities. The patient had not previously received inpatient treatment. Physical examination revealed an ROM of 0°–135° (extension–flexion) in the left elbow and 0°–145° in the right elbow. Restriction of external rotation was observed in the left elbow with palpated snapping. The bearing angles of the left and right elbows are 15° and 20°, respectively. Abnormal movements of the radial head could be palpated while flexing and extending both elbows. Plain radiographs of both elbow joints in anterior–posterior (AP) and lateral (LAT) views revealed an anterior dislocation of the radial head, dysplastic capitellum, and valgus deformity in the proximal ulna on both sides. In addition, the length of the radius and ulna (LRU) was measured for both sides, as well as the ratio of the length of radius to ulna (RLRU) (Figure 1). Based on these findings, bilateral anterior CRHD was diagnosed. A visual analog scale (VAS) pain questionnaire was 7 reported to the patient for his left elbow during motion. Since the patient found a snapping sound, abnormal movement of the radial head, and exacerbated pain in his left elbow, which had deeply affected his quality of life, and after thoroughly discussing all treatment options, the surgical approach was preferred.

**Figure 2:**
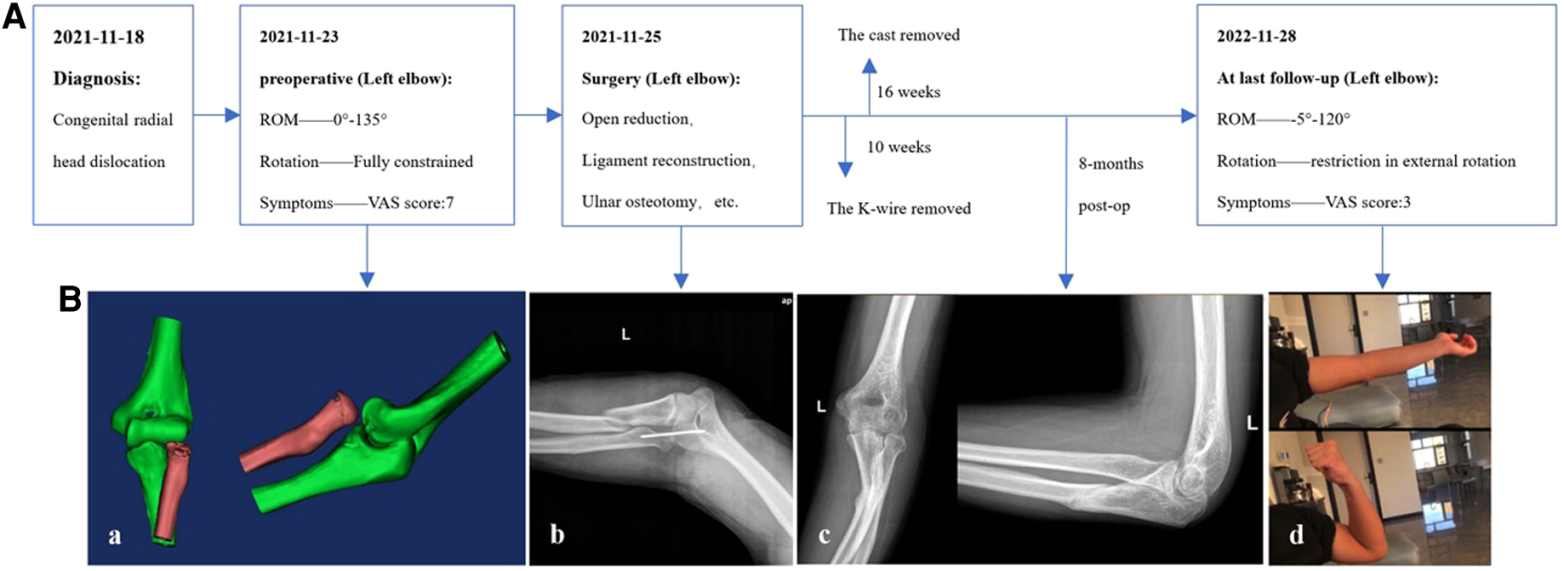
(**A**) Timeline of duration of each treatment. (**B**) (a) Preoperative CT scan and reconstruction of the left elbow revealed the dislocated radial head. (b) AP of intraoperative plain radiograph: the capitulum and radial head were secured by inserting a 2.5-mm K-wire while the elbow was flexed at a 90°; (c) Eight-month postoperative plain radiograph demonstrated callus formation at the proximal ulna, and the radial head was in its anatomic location; (d) After the surgery, the left elbow's ROM was assessed 1 year later. AP, anterior–posterior; ROM, range of motion; K-wire, Kirschner wire.

An extended lateral approach was used to expose the proximal radial and ulna. Proliferating scar tissues in the humeroradial joint were removed, and the protruding dome-shaped articular surface of the radial head was trimmed into a normal shape to fit the intended anatomical joint space. Oblique osteotomy was performed in the proximal ulnar metaphysis with an approximately 30° tilt to the shaft axis. By folding the ulnar shaft, the space for containing the radius was created, and the radial head was reduced to its anatomic position. The capitulum and radial head were then fixed with a 2.5-mm Kirschner wire (K-wire) with elbow flexion at 90°. The annular ligament was reconstructed to maintain the stability of the radial head over the long term. Postoperatively, indomethacin was administered at 25 mg/day for 6 weeks to prevent ectopic ossification (8). The elbow was placed in a cast at 90° flexion and in neutral position for 16 weeks. After 10 weeks, the K-wire was removed, and intermittent passive motion exercises were initiated. The cast was removed 16 weeks after surgery, and detailed active exercises were provided. Currently, the patient has been followed-up for 1 year and is still undergoing systematic rehabilitation. The ROM of the left elbow was now −5° to 120° (extension–flexion), and the external rotation was improved, but restriction still existed. At the 1-year postoperative follow-up, the VAS score had decreased from 7 to 3. Abnormal movement and snapping of the radial head were resolved. The entire treatment process was organized into a timeline, with corresponding photographs added at each stage of the process (Figure 2). Further guidance regarding functional exercises was provided to the patient.

## Literature review

### Materials and methods

Relevant literature published until December 2022 was retrieved from PubMed, Web of Science, and Embase databases. The keywords used for the searches included “congenital/connatural,” “radial head*,” “dislocation*,” and “adults.” The retrievals were only for articles written in English. The search field was the Title/Abstract. In addition, we screened the references of each study to ensure that most of the studies were included.

### Results

**Figure 3:**
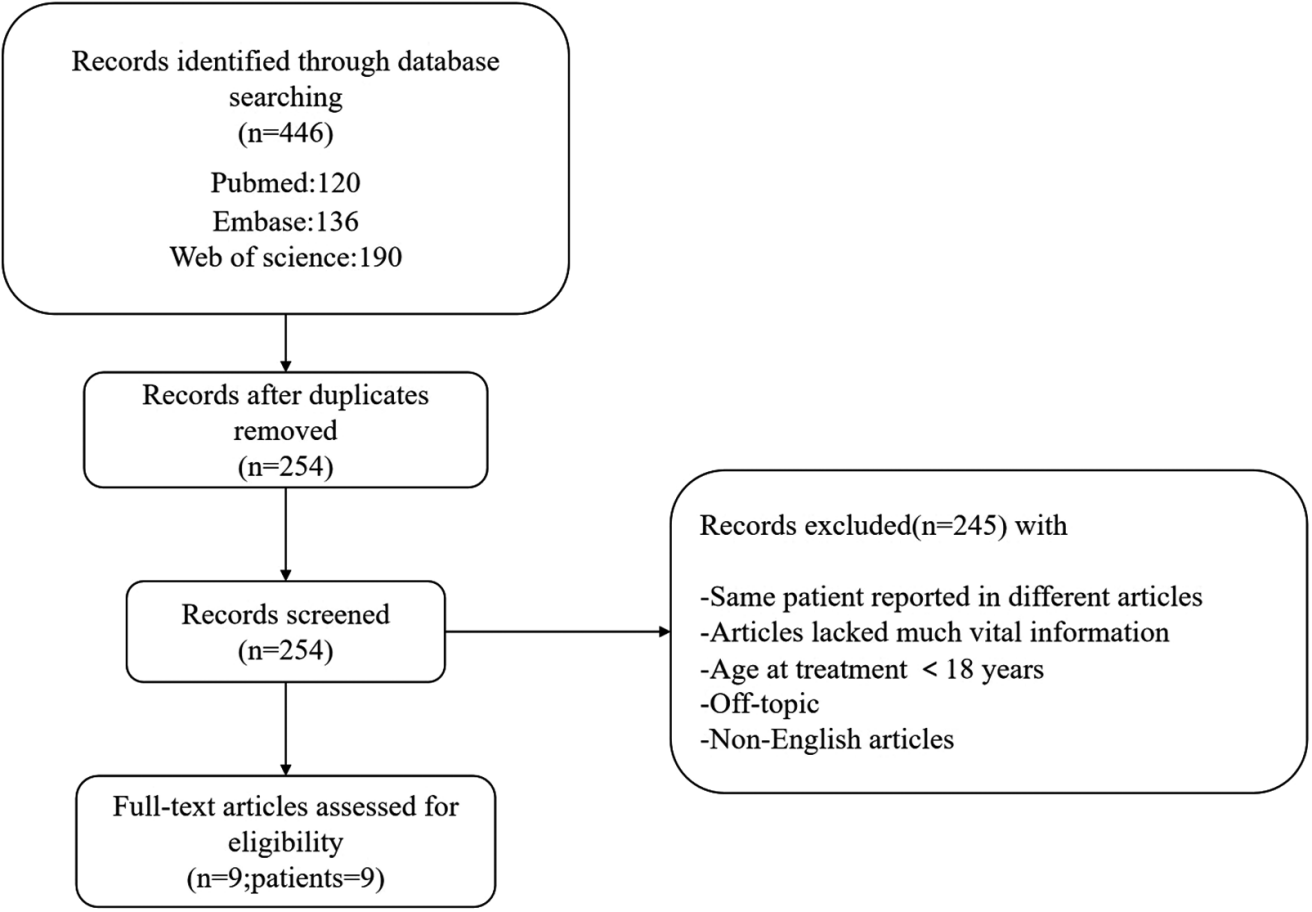
Flow diagram for the retrieval and screening of the reviewed publications.

In total, 446 studies were identified. After filtering out duplicates and off-topic and non-English articles, we retrieved a total of nine publications with nine CRHD patients older than 18 years. The literature search process is shown in Figure 3, and detailed information on the retrieved cases is listed in Table 1. Although some patients were diagnosed at the age of <18 years, no patients received any kind of treatment before adulthood. Of the nine patients, five had bilateral CRHD, two had unilateral CRHD, and no description on the sides was found in the other two patients.

**Table 1:** Clinical characteristics of retrieved cases.

\Author	Year	Age at diagnosis, years	Diagnosis	Sex	Side of surgery	Age at treatment, years	Treatments	Follow-up, months	Outcomes
Magee (9)	1947	27	CRHD (bilateral)	Male	NA	NA	NA	NA	NA
Futami et al. (10)	1992	19	CRHD (NA)	Male	NA	19	Rotation osteotomy of the radius	NA	Persistent limited forearm supination (30°)
Echtler and Burckhardt (11)	1997	45	CRHD (bilateral)	Female	NA	NA	NA	NA	NA
Bengard et al. (3)	2012	17	CRHD (NA)	NA	Right	18	Radial head excision, wrist arthroscopy and triangular fibrocartilage complex debridement	84	VAS: 8 (pre-op) to 1 (post-op)
Błoński et al. (12)	2012	17	CRHD (bilateral)	Male	Right	18	Resection of the proximal radius and partial anterior capsulectomy of the elbow joint	5	Flexion: 150 (pre-op) to 150 (post-op); Extension: 30 (pre-op) to 15 (post-op); Supination: 50 (pre-op) to 50 (post-op); Pronation: 10 (pre-op) to 70 (post-op)
Rosenbaum et al. (13)	2014	18	CRHD (unilateral)	Male	Left	NA	NA	NA	NA
Jones et al. (14)	2016	23	CRHD (bilateral)	Female	NA	NA	NA	NA	NA
Glener et al. (15)	2016	NA	CRHD (bilateral)	Female	Left	66	NA	NA	NA
Özdemir et al. (2)	2020	20	CRHD (unilateral)	Male	Left	NA	NA	NA	NA

NA, not available; CRHD, congenital dislocation of the radial head.

### Etiology and diagnosis

According to the medical histories of the reviewed cases, two patients were found to have CRHD when they presented to the hospital with trauma; one patient who had Steinfeld syndrome was unable to pronate or supinate whose elbows on physical examination were diagnosed with bilateral CRHD; one patient with carpal tunnel syndrome was found to have bilateral CRHD; and another patient with radial longitudinal deficiency was found to have unilateral CRHD. Because of the possibility of inheriting factors, one patient was diagnosed with bilateral CRHD during her health check-up, while her daughter was also diagnosed with bilateral CRHD, but no clear genetic pattern was revealed. The diagnoses of the other three patients were made when they visited hospitals for pain or restriction of forearm rotation on their elbows.

### Treatment

There is no consensus on guidelines for treating adult CRHD. Among the patients included in our review, six individuals did not receive surgical treatment due to their lack of apparent pain or restricted movement, absence of elbow joint instability, personal preference against surgery, or having missed the optimal treatment age window for addressing this deformity. Out of the remaining patients, three opted for surgical intervention, which was performed either unilaterally or bilaterally based on the severity of their symptoms and the anticipated functional improvement.

## Discussion

Approximately 60% of patients with congenital dislocation of the radial head also have other congenital abnormalities of the upper limb (16, 17). CRHD cases may be signified by the following characteristics (1, 18): (i) bilateral involvement, (ii) concurrent with other congenital anomalies, (iii) familial occurrence, (iv) no history of trauma, (v) irreducibility by closed maneuver, and (vi) dislocation at birth. CRHD is relatively tolerable in patients with fewer symptoms or intact ROM in the elbow, since quality of life is not significantly affected in these cases. In the long run, however, complications such as elbow instability and radioulnar joint disorder may occur in addition to osteoarthritis, neuropathy, or carpal tunnel syndrome (4, 19–21). According to Mardam-Bey and Ger (18), all patients without other anomalies were bilateral. However, much lower incidences of bilateral cases were reported by Almquist et al. (22) (42% of all cases) and Miura (23) (11 out of 34 cases). No difference in diagnostic standards has been reported for adult or juvenile CRHD.

However, the treatment strategy for adult patients with CRHD remains controversial. In our review, Futami et al. (10) performed rotational osteotomy of the radius; Bengard et al. (3) performed radial head excision, wrist arthroscopy, and triangular fibrocartilage complex debridement; and Błoński et al. (12) combined proximal radius osteotomy and partial anterior capsulectomy of the elbow joint. Despite limited improvement in pain, the overall outcomes were not ideal. One patient complained of persistent limitation in forearm supination (30°), and another patient did not show any improvement in function. As symptoms in childhood are usually mild and many remain asymptomatic during adolescence, follow-up without intervention is the standard management (1, 5, 24). Most authors favor conservative treatment or observation (1, 12, 18, 25). In some cases of CRHD diagnosed in childhood, closed reduction is still possible in selected cases with severe impairment of upper limb function or pain (12, 26). Radial head resection is indicated for adults but not for children (27, 28). Radial head resection can be performed in skeletally mature patients (1, 13), but Almquist et al. (22) were concerned about the loss of stability and development of cubitus valgus after the operation in the long term. Rotational osteotomy of the radius is a surgical procedure based on the principle of functional anatomy, according to which the radial head attains its best stability when the forearm is externally rotated (10). This procedure reduces the tension of the biceps tendon, which is considered the main factor responsible for recurrent anterior dislocation. It can further increase the strength of the pronator teres and pronator quadratus muscles. According to the literature, ulna osteotomy can also be applied in pediatric ulna deformity cases with favorable outcomes (5, 29), but it should be weighing against the potential risks of progressive cubitus valgus, supination weakness, and limitation in functional improvement in adult cases (11, 18, 24).

In the current case, the patient had bilateral anterior dislocations without any history of trauma. The anterior dislocation and overgrowth of the proximal radius may have resulted in an increased pressure or repetitive minor trauma on the lateral epiphysis of humerus, which eventually resulted in growth arrest, and with the continuing growth of the medial epiphysis, an additional cubitus valgus was brought (1). The bilateral radial heads of our patient were dome-shaped and dysplastic, and the nature of such anatomic characters indicated its congenital origin (30). Congenital malformation can cause length deformity of the radius or ulna; the real LRU is not accurate due to the magnification scale of our radiographs, and the normal RLRU ranged from 0.9286 to 0.9508 AP, and 0.9579 to 0.9698 LAT (31), which mismatch with our patient. Consequently, based on the presence of bilateral involvement, symmetrical radiographic findings, and the absence of a history of trauma, a posttraumatic dislocation has been ruled out as a potential cause for the patient's condition (7). Surgical intervention is recommended for the same reasons as correction of cubitus varus deformity, including functional limitations, elbow pain, and cosmetic concerns (1). The exacerbated pain and palpated snapping during motion in the left elbow reported by the patient were indications for surgery. Since postoperative complications such as elbow stiffness, ulnar nerve damage, and persistent malformation are common (1), the advantages and disadvantages of the surgery were explained to the patient in detail. The specific causes of CRHD remain unclear. Although familial occurrence has been reported, the exact genetic pattern has been confirmed (11, 32). Posterior dislocation is the most common form of this condition (65%), followed by anterior dislocation (18%) and lateral dislocation (17%) (1, 18). Open reduction combined with radius and/or ulnar osteotomy has been reported to produce favorable results in young patients (1, 5, 7, 29, 30, 33). This method not only prevents elbow instability that may occur after radial head resection but also treats the ulnar valgus deformity. We decided to perform these procedures along with temporary fixation of the radial head with a K-wire, even though our patient was older than those in the literature. Moreover, annular ligament reconstruction was applied to maintain the radial head in an anatomical position in the long term. Although the results of the surgery may not be perfect, the patient still had occasional pain; the restriction in external rotation was not fully restored, but the instability of the elbow during flexion was resolved. Therefore, we recommend that the patient continue aggressive functional exercise to increase the ROM of the elbow under the guidance of an experienced rehabilitation doctor.

## Conclusion

In the current study, we reviewed the etiology, diagnosis, and treatment strategies of adult CRHD and presented an adult case of bilateral CRHD with exacerbated pain and snapping during motion on one side. Based on the knowledge in the literature and our experiences, we believe that the primary indications for surgery are excessive pain, apparent instability, and abnormal movement in the elbow, which affect the quality of life. We do not recommend surgical treatment for the sole purpose of motion improvement, as well as for asymptomatic cases.

## Data Availability

The original contributions presented in the study are included in the article, further inquiries can be directed to the corresponding authors.

## References

[1] Kaas L Struijs PA. Congenital radial head dislocation with a progressive cubitus valgus: a case report. Strategies Trauma Limb Reconstr. (2012) 7(1):39–44. 10.1007/s11751-011-0126-z 22223165 PMC3332326

[2] Özdemir M Kavak RP Akdağ M Güven S. An unusual phenotype of radial longitudinal deficiency (radial hemimelia) presenting in a young adult male. Radiol Case Rep. (2020) 15(6):741–4. 10.1016/j.radcr.2020.02.023 32308776 PMC7155002

[3] Bengard MJ Calfee RP Steffen JA Goldfarb CA. Intermediate-term to long-term outcome of surgically and nonsurgically treated congenital, isolated radial head dislocation. J Hand Surg Am. (2012) 37(12):2495–501. 10.1016/j.jhsa.2012.08.032 23123151

[4] Lahoti O Akilapa O. Not kidding! Sequalae of elbow trauma in children. J Clin Orthop Trauma. (2021) 20:101471. 10.1016/j.jcot.2021.06.001 34194970 PMC8217682

[5] Jie Q Liang X Wang X Wu Y Wu G Wang B. Double ulnar osteomy for the treatment of congenital radial head dislocation. Acta Orthop Traumatol Turc. (2019) 53(6):442–7. 10.1016/j.aott.2019.08.010 31540774 PMC6938992

[6] Maruyama M Takahara M Kikuchi N Ito K Watanabe T Ogino T. Snapping elbow with congenital radial head dislocation: case report. J Hand Surg Am. (2010) 35(6):981–5. 10.1016/j.jhsa.2010.02.026 20456870

[7] Yamazaki H Kato H. Open reduction of the radial head with ulnar osteotomy and annular ligament reconstruction for bilateral congenital radial head dislocation: a case with long-term follow-up. J Hand Surg Eur. (2007) 32(1):93–7. 10.1016/j.jhsb.2006.09.003 17129644

[8] Bochat K Mattin AC Ricciardo BJ. The efficacy of nonsteroidal anti-inflammatories in the prevention of heterotopic ossification following elbow trauma surgery. JSES Int. (2021) 5(4):793–6. 10.1016/j.jseint.2021.04.004 34223432 PMC8245983

[9] Magee RK. Bilateral congenital dislocation of radial head. Lancet. (1947) 1(6451):519. 10.1016/S0140-6736(47)91628-0 20294840

[10] Futami T Tsukamoto Y Fujita T. Rotation osteotomy for dislocation of the radial head. 6 cases followed for 7 (3-10) years. Acta Orthop Scand. (1992) 63(4):455–6. 10.3109/17453679209154767 1529702

[11] Echtler B Burckhardt A. Isolated congenital dislocation of the radial head. Good function in 4 untreated patients after 14-45 years. Acta Orthop Scand. (1997) 68(6):598–600. 10.3109/17453679708999034 9462364

[12] Błoński M Podgórski A Zakrzewski P Pomianowski S. Surgical management of congenital radial head dislocation: a case report. Ortop Traumatol Rehabil. (2012) 14(4):385–91. 10.5604/15093492.1005087 23043061

[13] Rosenbaum AJ Leonard G Uhl RL Mulligan M Bagchi K. Congenital posterior dislocation of the radial head. Orthopedics. (2014) 37(1):11. 10.3928/01477447-20131219-01 24410301

[14] Jones GE Robertson L Maniyar A Shammas C Phelan MM Vasudevan PC Microform holoprosencephaly with bilateral congenital elbow dislocation; increasing the phenotypic spectrum of Steinfeld syndrome. Am J Med Genet A. (2016) 170(3):754–9. 10.1002/ajmg.a.37511 26728615

[15] Glener AD Gandolfi B Desai MJ Ruch DS. Median nerve compression by radial head osteophyte. J Hand Surg Am. (2016) 41(12):e481–3. 10.1016/j.jhsa.2016.08.016 27663050

[16] Kelly DW. Congenital dislocation of the radial head: spectrum and natural history. J Pediatr Orthop. (1981) 1(3):295–8. 10.1097/01241398-198111000-00009 7334108

[17] Karuppal R Marthya A Raman RV Somasundaran S. Case report: congenital dislocation of the radial head -a two-in-one approach. F1000Res. (2014) 3:22. 10.12688/f1000research.3-22.v1 27158442 PMC4857746

[18] Mardam-Bey T Ger E. Congenital radial head dislocation. J Hand Surg Am. (1979) 4(4):316–20. 10.1016/S0363-5023(79)80067-2 469206

[19] Austin R. Tardy palsy of the radial nerve from a Monteggia fracture. Injury. (1976) 7(3):202–4. 10.1016/0020-1383(76)90213-8 1254333

[20] Tawse B J A. The treatment of malunited anterior Monteggia fractures in children. J Bone Joint Surg Br. (1965) 47(4):718–23. 10.1302/0301-620X.47B4.718 5846773

[21] Ha T Grant S Huntley JS. Monteggia type IV fracture in a child with radial head dislocation irreducible by closed means: a case report. BMC Res Notes. (2014) 7:539. 10.1186/1756-0500-7-539 25129627 PMC4150939

[22] Almquist EE Gordon LH Blue AI. Congenital dislocation of the head of the radius. J Bone Joint Surg Am. (1969) 51(6):1118–27. 10.2106/00004623-196951060-00007 5306675

[23] Miura T. Congenital dislocation of the radial head. J Hand Surg Br. (1990) 15(4):477–81. 10.1016/0266-7681(90)90095-L 2269842

[24] Pokall S Sterniste W Pärtan G Hartmann B Girsch W. Elbow luxation at birth? Differential diagnosis of elbow disorders presenting at birth. Z Geburtshilfe Neonatol. (2019) 223(4):239–44. 10.1055/a-0891-1168 31096277

[25] Kuminack KF Reising K Schwering L Suedkamp NP Strohm RC. Congenital radial head dislocation on both sides. Unfallchirurg. (2007) 110(2):171–5. 10.1007/s00113-006-1158-6 17058062

[26] Sferopoulos NK Anagnostopoulos D. Anterior dislocation of the elbow in a child with congenital posterior dislocation of the radial head. A case report. Acta Orthop Belg. (1999) 65(3):378–81. . 10546364

[27] Koulali-Idrissi K Rafai M Largab A Trafeh M. Isolated dislocation of the radial head in an adult (case report and literature review). Chir Main. (2005) 24(2):103–5. 10.1016/j.main.2005.01.002 15861980

[28] Mizuno K Usui Y Kohyama K Hirohata K. Familial congenital unilateral anterior dislocation of the radial head: differentiation from traumatic dislocation by means of arthrography. A case report. J Bone Joint Surg Am. (1991) 73(7):1086–90. 10.2106/00004623-199173070-00020 1874773

[29] Liu R Miao W Mu M Wu G Qu J Wu Y. Ulnar rotation osteotomy for congenital radial head dislocation. J Hand Surg Am. (2015) 40(9):1769–75. 10.1016/j.jhsa.2015.06.005 26198841

[30] Kim HT Conjares JNV Suh JT Yoo CI. Chronic radial head dislocation in children, part 1: pathologic changes preventing stable reduction and surgical correction. J Pediatr Orthop. (2002) 22(5):583–90. . 12198458

[31] Wu C Wang D Mo Y Zhang Z Ning B. Characteristics of the length of the radius and ulna in children. Front Pediatr. (2022) 10:737823. 10.3389/fped.2022.737823 36016874 PMC9395915

[32] Gattey PH Wedge JH. Unilateral posterior dislocation of the radial head in identical twins. J Pediatr Orthop. (1986) 6(2):220–1. 10.1097/01241398-198603000-00018 3958177

[33] Kim HT Park BG Suh JT Yoo CI. Chronic radial head dislocation in children, part 2: results of open treatment and factors affecting final outcome. J Pediatr Orthop. (2002) 22(5):591–7. . 12198459

